# Supplementary material for: How did the urban and rural resident basic medical insurance integration affect medical costs?—Evidence from China
Source: PLoS One. 2025 Jul 18;20(7):e0325614. doi: 10.1371/journal.pone.0325614 (PMC12274002; doi:10.1371/journal.pone.0325614)
Supplement: S1 Table — (DOCX) [file pone.0325614.s001.docx]

**S1 Table.** Income and Expenditure of NCMS and URBMI in 2016

|  | Funding channels | Funding levels | Reimbursement rate | Number of drugs reimbursed | Per capita fund expenditures |
| --- | --- | --- | --- | --- | --- |
| NCMS | Government subsidy of about 80%, individual contribution of about 20% | About 470 RMB | 75% | 1100 | 417 |
| URBMI | Government subsidy of about 70%, individual contribution of about 30% | About 980 RMB | 70% | 2900 | 629 |
