# Supplementary material for: How did the urban and rural resident basic medical insurance integration affect medical costs?—Evidence from China
Source: PLoS One. 2025 Jul 18;20(7):e0325614. doi: 10.1371/journal.pone.0325614 (PMC12274002; doi:10.1371/journal.pone.0325614)
Supplement: S2 Table — (DOCX) [file pone.0325614.s002.docx]

**S2 Table.** Policy changes after health insurance integration

| City | Policy changes after health insurance integration |
| --- | --- |
| Beijing | (1) Comprehensive improvement of medical insurance treatment: the maximum reimbursement rate for outpatient services has been increased by 5 percentage points, the maximum reimbursement rate for hospitalization has been increased by 5-10 percentage points, the reimbursement rate for major disease insurance has been increased by 10 percentage points, the annual hospitalization ceiling has been increased from 180,000 to 200,000, and the outpatient starting line has been lowered in hospitals of first-class and below for urban residents, while it remains unchanged for rural residents. In terms of reimbursement methods, before the integration, urban residents' medical insurance had realized real-time settlement for card-carrying medical treatment, while rural residents' medical treatment was first advanced by individuals and then reimbursed, and after the unification of urban and rural areas, real-time settlement was realized for all of them.3 In terms of financing, the per capita financial clothing was raised to RMB 1,430.4 In terms of fixed-point medical institutions and the following medical institutions, and in terms of the time limit for the validity of the referral, the types of medicines that can be reimbursed will be expanded from the current 2,510 to more than 3,000. |
| Xinjiang | 1. Medical insurance catalog: the range of medicines available to former New Rural Cooperative participants has expanded from less than 1,500 to nearly 3,000. 2. Medical insurance treatment: the reimbursement ratio for tertiary hospitals in the autonomous region has been increased. |
| Fujian Province | Fujian Province: 1. Medical insurance directory: implement the city's unified basic medical insurance drug directory, diagnostic and treatment item directory, and the scope and standard of medical service facilities, and mainly improve the former New Farmers' Cooperative's insurance directory. 2. designated institutions: adjust the coordinated outpatient clinics, and incorporate village health clinics meeting the conditions of integrated management into the designated points of medical insurance. 3. medical insurance treatment: improve the reimbursement treatment for hospitalization, and reduce the threshold of reimbursement of the major disease insurance from The reimbursement threshold for major disease insurance was lowered from 20,000 yuan to 15,000 yuan after deducting the reimbursement from the basic medical insurance; the reimbursement rate for major diseases was raised, and the reimbursement rate for nine major diseases, including childhood leukemia, was uniformly raised to 80%. The reimbursement rate for severe mental illness has been increased from 80% to 90%, and the annual reimbursement ceiling for 17 chronic diseases such as diabetes has been raised.4. Focus on the poor: financial support for the poor has been strengthened after illnesses, and the reimbursement rate for the poor with documented cards has been raised to more than 96% of the total amount of regular medical expenses incurred in the current year.5. Enhancement of the quality of services: health insurance service stations have been set up in the city's second-rate and designated hospitals to facilitate the public's access to health care. to facilitate the public's access to medical care. |
| Nanjing, Jiangsu Province | 1. Health insurance directory: the original new rural cooperative health insurance directory coverage is narrow, about 1,000 kinds of drugs, the original residents of the health insurance directory is relatively wide, involving a total of 2,800 kinds of drugs, diagnosis and treatment and medical consumables 66,000, fixed-point medical institutions in the city of nearly 850, since 2017, the implementation of a unified urban and rural residents of the health insurance directory of drugs, diagnosis and treatment items (including special medical materials), medical service facilities. 2, health insurance treatment: to hospitalization outside the area of the starting line from 1200 yuan to 1,000 yuan. 3, health insurance financing: the financing of residents of the former New rural Cooperative not classified by population groups. Medical insurance treatment: the starting line for hospitalization in designated hospitals outside the district was lowered from 1,200 yuan to 1,000 yuan. 3, health insurance financing: the original New Rural Cooperative residents' financing was not classified according to the population, and after the unification of the different identities of the insured people as seniors, students, children and corpses, etc., the implementation of differentiated contributions. |
| Tianjin | 1. Health insurance financing: urban and rural residents have broken down the restrictions on household registration and unified the "one system, multiple grades" payment system. 2. Health insurance directory: the scope of fixed-point hospitals, medication directory, diagnosis and treatment items directory and service facilities directory for rural residents have been shared with those of employees' health insurance, and the number of fixed-point service organizations that they can choose from has been expanded to more than 1,600, with more than 7,000 kinds of medicines reimbursed. In terms of medical insurance treatment, the reimbursement ratio has been increased. Outpatient reimbursement for rural residents within the scope of the residents' anaerobic insurance policy has increased from nothing to something, and the reimbursement ratio has increased by more than 30 percentage points compared with that of the New Rural Cooperative Medical Care System, with the maximum payment limit raised from 30,000 yuan to 180,000 yuan on average.4. Payment methods: All township health centers in the city have been included in the medical insurance payment system, and have realized card-based and networked settlement of medical bills. |
| Henan Province | 1. In terms of medical insurance treatment: adjusting the starting line for urban residents' and New Rural Cooperative's major disease insurance from 18,000 yuan and 15,000 yuan respectively to 15,000 yuan; increasing the annual maximum payment limit for major disease insurance from 300,000 yuan to 400,000 yuan. 2. In terms of the medical insurance catalog: incorporating into the catalogue of medical insurance items that are not covered by the urban medical insurance and the New Rural Cooperative but which are clinically necessary, especially high-cost medical services for the treatment of thousands of critically ill patients, such as liver transplants, corrective scoliosis surgery and 88 items related to paediatrics. 3. In terms of medical institutions, the former urban residents' anaesthesia insurance and New Agricultural Cooperative Medical Care designated anaesthesia institutions were integrated into the scope of fixed-point management of urban and rural residents' medical insurance. |
