# Supplementary material for: How did the urban and rural resident basic medical insurance integration affect medical costs?—Evidence from China
Source: PLoS One. 2025 Jul 18;20(7):e0325614. doi: 10.1371/journal.pone.0325614 (PMC12274002; doi:10.1371/journal.pone.0325614)
Supplement: S3 Table — (DOCX) [file pone.0325614.s003.docx]

**S3 Table.** Duration of urban and rural residents' health insurance integration by city

| **Province** | **City** | **Time** | **Province** | **City** | **Time** | **Province** | **City** | **Time** |
| --- | --- | --- | --- | --- | --- | --- | --- | --- |
| Yunnan | Baoshan | 2017 | Jiangsu | Suzhou | 2012 | Guangxi | Hechi | 2017 |
|  | Chuxiong | 2017 |  | Xuzhou | 2018 |  | Nanning | 2017 |
|  | Zhaotong | 2017 |  | Taizhou | 2018 |  | Yulin | 2017 |
|  | Kunming | 2017 |  | Yancheng | 2018 |  | Guilin | 2017 |
|  | Lincang | 2017 |  | Suqian | 2018 | Anhui | Bozhou | 2017 |
|  | Lijiang | 2017 |  | Yangzhou | 2018 |  | Fuyang | 2017 |
| Fujian | Zhangzhou | 2018 |  | Lianyungang | 2018 |  | Huainan | 2017 |
|  | Putian | 2013 | Chongiqng | Chongqing | 2012 |  | Anqing | 2017 |
|  | Fuzhou | 2016 | Gansu | Lanzhou | 2018 |  | suzhou | 2017 |
|  | Ningde | 2015 |  | Dingxi | 2019 |  | Lu'an | 2017 |
| Qinghai | Haidong | 2016 |  | Pingliang | 2019 | Hubei | Chaohu | 2017 |
| Sichuan | Guang'an | 2017 |  | Zhangye | 2018 |  | Xiangfan | 2022 |
|  | Chengdu | 2009 | Heilongjiang | Jixi | 2018 |  | Enshi Tujia and Miao Autonomous Prefecture | 2018 |
|  | Liangshan Yi Autonomous Prefecture | 2018 |  | Qiqihar | 2016 |  | Huanggang | 2020 |
|  | Nanchong | 2017 |  | Harbin | 2018 | Shannxi | Jingmen | 2017 |
|  | Yibin | 2017 |  | Jiamusi | 2018 |  | Weinan | 2020 |
|  | Ziyang | 2021 |  | Foshan | 2017 |  | Baoji | 2020 |
|  | Neijiang | 2022 | Guangdong | Shenzhen | 2018 |  | Yulin | 2020 |
|  | Ganzi Tibetan Autonomous Prefecture | 2016 |  | Chaozhou | 2012 | Shandong | Hanzhong | 2022 |
|  | Mianyang | 2017 |  | Guangzhou | 2015 |  | Binzhou | 2014 |
|  | Meishan | 2015 |  | Maoming | 2012 |  | Qingdao | 2015 |
| Hebei | Baoding | 2018 |  | Qingyuan | 2011 |  | Jinan | 2022 |
|  | Shijiazhuang | 2017 |  | Jiangmen | 2010 |  | Zaozhuang | 2014 |
|  | Cangzhou | 2017 | Liaoning | Benxi | 2020 |  | Linyi | 2014 |
|  | Chengde | 2017 |  | Anshan | 2020 |  | Liaocheng | 2014 |
| Jiangxi | Jingdezhen | 2018 |  | Dalian | 2020 |  | Weihai | 2014 |
|  | Nanchang | 2016 |  | Chaoyang | 2020 |  | Weifang | 2014 |
|  | Yichun | 2017 |  | Jinzhou | 2019 | Henan | Dezhou | 2014 |
|  | Shangrao | 2017 | Shanxi | Yangquan | 2018 |  | Anyang | 2017 |
|  | Jiujiang | 2017 |  | Xinzhou | 2017 |  | Luoyang | 2021 |
|  | Ji'an | 2017 |  | Yuncheng | 2017 |  | Zhoukou | 2017 |
|  | Ganzhou | 2017 |  | Linfen | 2017 |  | Zhengzhou | 2017 |
| Xijiang | Aksu Area | 2018 | Shanghai | Shanghai | 2016 |  | Puyang | 2017 |
| Beijing | Beijing | 2018 | Tianjin | Tianjin | 2009 |  | Pingdingshan | 2017 |
| Neimenggu | Xilin Gol League | 2018 | Hunan | xinyang | 2017 |  | Jiaozuo | 2017 |
|  | Hohhot | 2017 |  | Shaoyang | 2017 | Zhejiang | Huzhou | 2016 |
|  | Xing'anmeng | 2017 |  | yueyang | 2017 |  | Ningbo | 2016 |
|  | Chifeng | 2017 |  | Changsha | 2017 |  | Hangzhou | 2011 |
|  | Hulunbeier | 2017 |  | Yiyang | 2017 |  | Lishui | 2014 |
| Jilin | Jilin | 2020 |  | Loudi | 2017 |  | Jiaxing | 2004 |
|  | Siping | 2020 |  | Changde | 2017 |  | Taizhou | 2015 |
| Guizhou | Qiandongnan | 2021 |  |  |  |  |  |  |
|  | Qiannan | 2020 |  |  |  |  |  |  |

Note. Most cities will launch their URRBMI integration reform the year before the policy is implemented, while the actual year of implementation is at the beginning of the following year. CHARLS opens its surveys in the second half of each year, and data such reflect the current year's year.
