# Supplementary material for: How did the urban and rural resident basic medical insurance integration affect medical costs?—Evidence from China
Source: PLoS One. 2025 Jul 18;20(7):e0325614. doi: 10.1371/journal.pone.0325614 (PMC12274002; doi:10.1371/journal.pone.0325614)
Supplement: S4 Table — (DOCX) [file pone.0325614.s004.docx]

**S4 Table.** Meaning of variables

| Variables | Meaning of variables |
| --- | --- |
| Age | Age at time of interview |
| Sex | Female = 0, Male = 1 |
| Location | Rural=1, Urban=2 |
| Education | Elementary school = 1, Junior high school = 2, Senior high school = 3, University = 4, Postgraduate student = 5 |
| Marriage | Unmarried (including divorced and widowed) = 0, Married = 1 |
| Health Status | Very unhealthy = 1, Less unhealthy = 2, Fair = 3, Healthy = 4, Very healthy = 5 |
| Disability | No = 0, Yes = 1 |
| Physical activity | Whether walking for leisure, sport, exercise or recreation. No=0, Yes=1 |
| Smoking | No=0, Yes=1 |
| Drinking | No=0, Yes=1 |
| Medical Insurance | Urban workers' health insurance=1, urban residents' insurance=2, new rural cooperative=3, urban and rural residents=4, commercial insurance=5 Insurance |
| Health insurance | No=0, Yes=1 |
| Satisfaction with medical services | Satisfaction with local medical services, very dissatisfied=1, quite dissatisfied=2, average=3, quite satisfied=4, very satisfied=5 |
| Regular medical checkups | No=0,Yes1 |
| Outpatient visits | Whether outpatient visit in the past month. No=0, Yes=1 |
| Type of Outpatient | General hospitals (i.e. general hospitals, excluding Chinese medicine hospitals) =1, Specialized hospitals (excluding Chinese medicine hospitals) =2, Chinese medicine hospitals=3, Community health service centers=4, Township health centers=5, Health service stations=6, Village clinics/private clinics=7, Nursing institutions=8 |
| Outpatient Costs | Cost of last outpatient visit (yuan) |
| Outpatient OOP Costs | Out-of-pocket expenses for last outpatient visit (yuan) |
| Inpatient visits | Whether hospitalization visit in the past year. No=0, Yes=1 |
| Inpatient Costs | Cost of last hospitalization (yuan) |
| Inpatient OOP Costs | Out-of-pocket expenses spent on last hospitalization (yuan) |
| Type of inpatient | General hospitals (i.e. general hospitals, excluding Chinese medicine hospitals)=1,Specialized hospitals (excluding Chinese medicine hospitals)=2,Chinese medicine hospitals=3,Community health service centers=4,Township health centers=5,Health service stations=6,Village clinics/private clinics=7,Nursing institutions=8 |
| Distance to medical institutions | Distance of last hospitalization. km |
| Total medical expenditure | Respondent's family's direct or indirect treatment expenditure in the past year, indirect medical expenditure, refers to the transportation cost, nutritional cost, family accompanying cost, etc. because of medical treatment (yuan) |
| Family size | Number of people in the respondent's family (persons) |
| Total annual wage income | Respondent's wage income in the past year (yuan) |
| Annual income from self-employment | Respondent's income from self-employment in the past year (yuan) |
| Government Pension income | Respondent's pension income acquired in the past year (yuan) |
| Resident pension income | Respondent's pension income acquired in the past year (yuan) |
| Commercial Pension | Pension income acquired by respondent in past year (yuan) |
| Other pensions | Other pension income acquired by the respondent in the past year (yuan) |
| Agriculture and forestry income | Respondent's income from farming and forestry activities acquired in the past year (yuan) |
| Livestock Income | Respondent's income from livestock activities in the past year (yuan) |
| Agricultural income | Respondent's income from farming activities in the past year (yuan) |
| Business income | Respondent's income from self-employment industry in the past year (yuan) |
