# Supplementary material for: How did the urban and rural resident basic medical insurance integration affect medical costs?—Evidence from China
Source: PLoS One. 2025 Jul 18;20(7):e0325614. doi: 10.1371/journal.pone.0325614 (PMC12274002; doi:10.1371/journal.pone.0325614)
Supplement: S5 Table — (DOCX) [file pone.0325614.s005.docx]

**S5 Table.** Impact of URRBMI on healthcare resource utilization and medical costs-DID

|  | Outpatient visits | Inpatient visits | Outpatient OOP costs | Inpatient OOP costs | Medical expenditure |
| --- | --- | --- | --- | --- | --- |
| DID | 0.000 | 0.019^***^ | 0.152^**^ | -0.001 | 0.261 |
|  | (0.001) | (0.005) | (0.074) | (0.086) | (0.551) |
| Age | 0.000 | 0.004^***^ | 0.006 | 0.010^**^ | -0.013 |
|  | (0.000) | (0.000) | (0.004) | (0.004) | (0.025) |
| Sex | 0.001 | 0.022^***^ | -0.019 | -0.046 | 0.284 |
|  | (0.001) | (0.006) | (0.082) | (0.091) | (0.607) |
| Marriage | 0.000 | -0.021^**^ | 0.187^*^ | 0.124 | 1.992^***^ |
|  | (0.002) | (0.008) | (0.102) | (0.103) | (0.601) |
| Regular medical checkups | 0.008^***^ | 0.049^***^ | -0.048 | -0.051 | -0.016 |
|  | (0.002) | (0.006) | (0.077) | (0.081) | (0.514) |
| Health Status | 0.000 | -0.072^***^ | -0.133^***^ | 0.216^***^ | -0.757^***^ |
|  | (0.000) | (0.002) | (0.040) | (0.072) | (0.246) |
| Disability | 0.052^***^ | 0.069^***^ | 0.210^*^ | -0.281^***^ | 0.439 |
|  | (0.005) | (0.010) | (0.118) | (0.102) | (0.936) |
| Drinking | 0.000 | -0.028^***^ | -0.265^***^ | -0.425^***^ | -0.186 |
|  | (0.001) | (0.006) | (0.097) | (0.121) | (0.522) |
| Smoking | -0.001 | -0.045^***^ | -0.112 | -0.495^***^ | -1.447^**^ |
|  | (0.002) | (0.007) | (0.127) | (0.138) | (0.630) |
| Income | 0.000 | 0.008^***^ | 0.112^***^ | 0.213^***^ | -2.326^***^ |
|  | (0.000) | (0.002) | (0.025) | (0.027) | (0.252) |
| _cons | -0.001 | 0.090^***^ | 5.253^***^ | 6.688^***^ | 10.528^***^ |
|  | (0.005) | (0.027) | (0.332) | (0.435) | (2.171) |
| N | 21047 | 21033 | 1710 | 1266 | 4676 |
| R-sq | 0.037 | 0.077 | 0.033 | 0.087 | 0.033 |

Note. ^*^, ^**^, ^***^ corresponding to p values ≤ 0.10, ≤ 0.05 and ≤ 0.01, respectively . 95% confidence interval reported in brackets.
