# Supplementary material for: How did the urban and rural resident basic medical insurance integration affect medical costs?—Evidence from China
Source: PLoS One. 2025 Jul 18;20(7):e0325614. doi: 10.1371/journal.pone.0325614 (PMC12274002; doi:10.1371/journal.pone.0325614)
Supplement: S6 Table — (DOCX) [file pone.0325614.s006.docx]

**S6 Table.** Impact of URRBMI on healthcare resource utilization and medical costs-DID with fixed time effects and fixed region effects

|  | Outpatient visits | Inpatient visits | Outpatient OOP costs | Inpatient OOP costs | Medical expenditure |
| --- | --- | --- | --- | --- | --- |
| DID | 0.000 | 0.019* | 0.221** | 0.247** | 1.678*** |
|  | (0.002) | (0.011) | (0.085) | (0.120) | (0.629) |
| Age | 0.000 | 0.004*** | -0.005 | -0.002 | -0.024 |
|  | (0.000) | (0.001) | (0.004) | (0.005) | (0.034) |
| Sex | 0.001 | 0.022** | 0.033 | 0.031 | 0.09 |
|  | (0.001) | (0.009) | (0.071) | (0.097) | (0.378) |
| Marriage | 0.000 | -0.022 | 0.113 | 0.116 | 1.980** |
|  | (0.002) | (0.016) | (0.113) | (0.115) | (0.856) |
| Regular medical checkups | 0.008*** | 0.050*** | 0.022 | 0.01 | 0.268 |
|  | (0.001) | (0.012) | (0.072) | (0.083) | (0.516) |
| Health Status | 0.000 | -0.073*** | -0.136*** | 0.242*** | -0.875** |
|  | (0.001) | (0.003) | (0.050) | (0.073) | (0.424) |
| Disability | 0.052*** | 0.070*** | 0.265** | -0.157* | 0.58 |
|  | (0.005) | (0.010) | (0.119) | (0.094) | (0.934) |
| Drinking | 0.000 | -0.028*** | -0.252** | -0.299*** | -0.13 |
|  | (0.001) | (0.009) | (0.108) | (0.106) | (0.540) |
| Smoking | -0.001 | -0.045*** | -0.087 | -0.351** | -1.161 |
|  | (0.002) | (0.011) | (0.125) | (0.158) | (0.739) |
| Income | 0.000 | 0.008 | 0.045* | 0.115*** | -2.449*** |
|  | (0.000) | (0.005) | (0.025) | (0.033) | (0.286) |
| Time effect | YES | YES | YES | YES | YES |
| Region effect | YES | YES | YES | YES | YES |
| _cons | -0.003 | 0.091 | 5.815*** | 8.447*** | 15.916*** |
|  | (0.006) | (0.069) | (0.410) | (0.496) | (2.840) |
| N | 21060 | 21046 | 1714 | 1272 | 4683 |
| R-sq | 0.038 | 0.078 | 0.141 | 0.228 | 0.044 |

Note. ^*^, ^**^, ^***^ corresponding to p values ≤ 0.10, ≤ 0.05 and ≤ 0.01, respectively . 95% confidence interval reported in brackets.
