# Supplementary material for: How did the urban and rural resident basic medical insurance integration affect medical costs?—Evidence from China
Source: PLoS One. 2025 Jul 18;20(7):e0325614. doi: 10.1371/journal.pone.0325614 (PMC12274002; doi:10.1371/journal.pone.0325614)
Supplement: S7 Table — (DOCX) [file pone.0325614.s007.docx]

**S7 Table.** PSM matching results test 1 (explanatory variable is outpatient OOP costs)

|  | Unmatched | Mean | %reduct | t-test | V(T)/ |  |  |  |
| --- | --- | --- | --- | --- | --- | --- | --- | --- |
| Variable | Matched | Treated | Control | %bias | bias | t | p>t | V(C) |
| Age | U | 61.72 | 60.84 | 9.70 |  | 1.74 | 0.08 | 1.05 |
|  | M | 61.72 | 61.79 | -0.70 | 92.60 | -0.16 | 0.87 | 1.00 |
| Sex | U | 0.36 | 0.33 | 7.40 |  | 1.33 | 0.18 | . |
|  | M | 0.36 | 0.37 | -1.10 | 85.10 | -0.25 | 0.80 | . |
| Marriage | U | 0.86 | 0.88 | -5.80 |  | -1.04 | 0.30 | . |
|  | M | 0.86 | 0.85 | 1.40 | 76.20 | 0.31 | 0.76 | . |
| Regular medical checkups | U | 0.31 | 0.30 | 1.60 |  | 0.29 | 0.77 | . |
|  | M | 0.31 | 0.29 | 4.20 | -163.70 | 0.98 | 0.33 | . |
| Health Status | U | 2.44 | 2.59 | -16.70 |  | -3.04 | 0.00 | 0.93 |
|  | M | 2.44 | 2.45 | -0.60 | 96.20 | -0.14 | 0.89 | 0.95 |
| Disability | U | 0.09 | 0.10 | -4.00 |  | -0.72 | 0.47 | . |
|  | M | 0.09 | 0.09 | 0.20 | 94.50 | 0.05 | 0.96 | . |
| Drinking | U | 0.21 | 0.15 | 15.30 |  | 2.70 | 0.01 | . |
|  | M | 0.21 | 0.22 | -1.90 | 87.60 | -0.41 | 0.68 | . |
| Smoking | U | 0.09 | 0.08 | 1.60 |  | 0.29 | 0.77 | . |
|  | M | 0.09 | 0.10 | -3.00 | -86.60 | -0.66 | 0.51 | . |
| Income | U | 3.25 | 3.48 | -16.00 |  | -2.85 | 0.00 | 1.17^*^ |
|  | M | 3.25 | 3.29 | -3.30 | 79.40 | -0.75 | 0.45 | 1.15^*^ |
