# Supplementary material for: How did the urban and rural resident basic medical insurance integration affect medical costs?—Evidence from China
Source: PLoS One. 2025 Jul 18;20(7):e0325614. doi: 10.1371/journal.pone.0325614 (PMC12274002; doi:10.1371/journal.pone.0325614)
Supplement: S8 Table — (DOCX) [file pone.0325614.s008.docx]

**S8 Table.** PSM matching results test 2 (explanatory variable is outpatient OOP costs)

| Samp le | Ps R^2^ | LRchi^2^ | p>chi^2^ | Mean Bias | Med Bias | B | R | %Var |
| --- | --- | --- | --- | --- | --- | --- | --- | --- |
| Unmatched | 0.02 | 29.38 | 0.00 | 8.10 | 6.60 | 30.4^*^ | 1.20 | 25.00 |
| Matched | 0.00 | 2.47 | 0.99 | 1.80 | 1.30 | 6.90 | 1.00 | 25.00 |
