# Supplementary material for: How did the urban and rural resident basic medical insurance integration affect medical costs?—Evidence from China
Source: PLoS One. 2025 Jul 18;20(7):e0325614. doi: 10.1371/journal.pone.0325614 (PMC12274002; doi:10.1371/journal.pone.0325614)
Supplement: S9 Table — (DOCX) [file pone.0325614.s009.docx]

**S9 Table.** PSM matching results test 1 (explanatory variable is inpatient OOP costs)

|  | Unmatched | Mean | %reduct | t-test | V(T)/ |  |  |  |
| --- | --- | --- | --- | --- | --- | --- | --- | --- |
| Variable | Matched | Treated | Control | %bias | bias | t | p>t | V(C) |
| Age | U | 65.46 | 65.36 | 1.00 | 0.14 | 0.89 | 0.88 |  |
|  | M | 65.49 | 65.10 | 4.00 | -294.80 | 0.69 | 0.49 | 0.90 |
| Sex | U | 0.40 | 0.39 | 1.20 | 0.16 | 0.88 | . |  |
|  | M | 0.40 | 0.39 | 1.20 | -0.70 | 0.20 | 0.84 | . |
| Marriage | U | 0.82 | 0.82 | 0.80 | 0.11 | 0.91 | . |  |
|  | M | 0.82 | 0.82 | -0.40 | 53.00 | -0.06 | 0.95 | . |
| Regular medical checkups | U | 0.42 | 0.36 | 11.20 | 1.48 | 0.14 | . |  |
|  | M | 0.41 | 0.44 | -5.90 | 46.80 | -0.99 | 0.32 | . |
| Health Status | U | 2.16 | 2.14 | 1.50 | 0.20 | 0.85 | 0.96 |  |
|  | M | 2.16 | 2.10 | 6.50 | -343.50 | 1.08 | 0.28 | 0.90 |
| Disability | U | 0.20 | 0.18 | 6.90 | 0.91 | 0.37 | . |  |
|  | M | 0.20 | 0.21 | -4.00 | 42.50 | -0.65 | 0.52 | . |
| Drinking | U | 0.16 | 0.10 | 17.60 | 2.26 | 0.02 | . |  |
|  | M | 0.16 | 0.15 | 1.60 | 91.10 | 0.25 | 0.81 | . |
| Smoking | U | 0.05 | 0.09 | -14.80 | -2.07 | 0.04 | . |  |
|  | M | 0.05 | 0.05 | -1.90 | 87.30 | -0.36 | 0.72 | . |
| Income | U | 3.13 | 3.09 | 2.40 | 0.32 | 0.75 | 0.99 |  |
|  | M | 3.12 | 3.18 | -3.80 | -58.70 | -0.64 | 0.52 | 0.99 |
