# Supplementary material for: How did the urban and rural resident basic medical insurance integration affect medical costs?—Evidence from China
Source: PLoS One. 2025 Jul 18;20(7):e0325614. doi: 10.1371/journal.pone.0325614 (PMC12274002; doi:10.1371/journal.pone.0325614)
Supplement: S11 Table — (DOCX) [file pone.0325614.s011.docx]

**S11 Table.** PSM matching results 1 (explanatory variable is medical expenditure)

|  | Unmatched | Mean | %reduct | t-test | V(T)/ |  |  |  |
| --- | --- | --- | --- | --- | --- | --- | --- | --- |
| Variable | Matched | Treated | Control | %bias | bias | t | p>t | V(C) |
| Age | U | 62.03 | 61.43 | 6.50 | 1.71 | 0.09 | 1.09^*^ |  |
|  | M | 62.03 | 62.18 | -1.60 | 74.90 | -0.70 | 0.48 | 1.07 |
| Sex | U | 0.44 | 0.45 | -2.60 | -0.70 | 0.49 | . |  |
|  | M | 0.44 | 0.43 | 2.40 | 10.20 | 1.02 | 0.31 | . |
| Marriage | U | 0.86 | 0.88 | -4.50 | -1.17 | 0.24 | . |  |
|  | M | 0.86 | 0.87 | -1.10 | 75.50 | -0.46 | 0.64 | . |
| Regular medical checkups | U | 0.28 | 0.26 | 4.40 | 1.16 | 0.25 | . |  |
|  | M | 0.28 | 0.28 | -0.10 | 96.80 | -0.06 | 0.95 | . |
| Health Status | U | 2.76 | 2.89 | -13.60 | -3.78 | 0.00 | 0.77^*^ |  |
|  | M | 2.76 | 2.73 | 2.90 | 78.50 | 1.30 | 0.19 | 0.84^*^ |
| Disability | U | 0.12 | 0.10 | 4.60 | 1.19 | 0.24 | . |  |
|  | M | 0.12 | 0.10 | 4.40 | 3.50 | 1.89 | 0.06 | . |
| Drinking | U | 0.26 | 0.24 | 5.20 | 1.36 | 0.18 | . |  |
|  | M | 0.26 | 0.25 | 1.90 | 63.50 | 0.80 | 0.42 | . |
| Smoking | U | 0.09 | 0.09 | 0.60 | 0.15 | 0.88 | . |  |
|  | M | 0.09 | 0.09 | -0.90 | -63.70 | -0.39 | 0.70 | . |
| Income | U | 3.17 | 3.43 | -17.70 | -4.72 | 0.00 | 0.96 |  |
|  | M | 3.17 | 3.17 | -0.10 | 99.60 | -0.03 | 0.97 | 0.94^*^ |
