# Supplementary material for: How did the urban and rural resident basic medical insurance integration affect medical costs?—Evidence from China
Source: PLoS One. 2025 Jul 18;20(7):e0325614. doi: 10.1371/journal.pone.0325614 (PMC12274002; doi:10.1371/journal.pone.0325614)
Supplement: S12 Table — (DOCX) [file pone.0325614.s012.docx]

**S12 Table.** PSM matching results 2 (explanatory variable is medical expenditure)

| Sample | Ps R^2^ | LRchi^2^ | p>chi^2^ | Mean Bias | Med Bias | B | R | %Var |
| --- | --- | --- | --- | --- | --- | --- | --- | --- |
| Unmatched | 0.01 | 42.71 | 0.00 | 6.40 | 4.50 | 24.40 | 0.90 | 50.00 |
| Matched | 0.00 | 9.73 | 0.47 | 1.80 | 1.80 | 7.20 | 1.00 | 50.00 |
