# Supplementary material for: How did the urban and rural resident basic medical insurance integration affect medical costs?—Evidence from China
Source: PLoS One. 2025 Jul 18;20(7):e0325614. doi: 10.1371/journal.pone.0325614 (PMC12274002; doi:10.1371/journal.pone.0325614)
Supplement: S13 Table — (DOCX) [file pone.0325614.s013.docx]

**S13 Table.** Impact of URRBMI integration on moral hazard

|  | Outpatient OOP costs | Inpatient OOP costs | Medical expenditure |
| --- | --- | --- | --- |
| DID | 0.237^***^ | 0.282^*^ | 0.384^***^ |
|  | (0.085) | (0.152) | (0.076) |
| Age | 0.000 | -0.005 | 0.004 |
|  | (0.005) | (0.006) | (0.003) |
| Sex | 0.024 | 0.043 | 0.027 |
|  | (0.076) | (0.108) | (0.042) |
| Marriage | 0.131 | 0.138 | 0.353^***^ |
|  | (0.120) | (0.142) | (0.079) |
| Regular medical checkups | 0.018 | -0.11 | 0.194^***^ |
|  | (0.077) | (0.110) | (0.055) |
| Health status | -0.136^***^ | -0.205^***^ | -0.263^***^ |
|  | (0.050) | (0.054) | (0.026) |
| Disability | 0.261^**^ | -0.184 | 0.134^**^ |
|  | (0.122) | (0.117) | (0.053) |
| Drinking | -0.253^**^ | -0.171 | -0.197^***^ |
|  | (0.115) | (0.130) | (0.056) |
| Smoking | -0.066 | -0.136 | -0.09 |
|  | (0.164) | (0.237) | (0.095) |
| Income | 0.075^***^ | 0.093^**^ | 0.030^*^ |
|  | (0.026) | (0.039) | (0.018) |
| Satisfaction with medical services | -0.094^***^ | -0.152^***^ | -0.051^*^ |
|  | (0.030) | (0.047) | (0.026) |
| Time effect | YES | YES | YES |
| Region effect | YES | YES | YES |
| _cons | 5.725^***^ | 10.233^***^ | 7.664^***^ |
|  | (0.451) | (0.518) | (0.244) |
| N | 1577 | 927 | 4593 |
| R-sq | 0.145 | 0.303 | 0.069 |

Note. ^*^, ^**^, ^***^ corresponding to p values ≤ 0.10, ≤ 0.05 and ≤ 0.01, respectively . 95% confidence interval reported in brackets.
