# Supplementary material for: How did the urban and rural resident basic medical insurance integration affect medical costs?—Evidence from China
Source: PLoS One. 2025 Jul 18;20(7):e0325614. doi: 10.1371/journal.pone.0325614 (PMC12274002; doi:10.1371/journal.pone.0325614)
Supplement: S14 Table — (DOCX) [file pone.0325614.s014.docx]

**S14 Table.** Impact of URRBMI integration on type of medical institutions and distance to medical institutions

|  | Outpatient type | Inpatient type | Distance to medical institutions |
| --- | --- | --- | --- |
| DID | 0.163^***^ | 0.049^*^ | 29.864^***^ |
|  | (0.049) | (0.025) | (9.072) |
| Age | -0.002 | 0.001 | -1.014^**^ |
|  | (0.003) | (0.001) | (0.465) |
| Sex | 0.075^**^ | 0.014 | -0.523 |
|  | (0.038) | (0.017) | (7.214) |
| Marriage | 0.076 | 0.003 | 11.798 |
|  | (0.048) | (0.026) | (8.711) |
| Regular medical checkups | 0.117^***^ | -0.018 | 2.961 |
|  | (0.031) | (0.019) | (8.232) |
| Health Status | 0.01 | -0.011 | -4.216 |
|  | (0.016) | (0.009) | (3.321) |
| Disability | 0.178^***^ | -0.001 | 8.197 |
|  | (0.032) | (0.031) | (13.266) |
| Drinking | -0.107^***^ | -0.046^*^ | -0.795 |
|  | (0.033) | (0.025) | (7.571) |
| Smoking | -0.172^***^ | -0.057 | 18.555^*^ |
|  | (0.058) | (0.037) | (9.463) |
| Income | 0.016 | 0.016^**^ | -1.875 |
|  | (0.013) | (0.007) | (2.055) |
| Time effect | YES | YES | YES |
| Region effect | YES | YES | YES |
| _cons | 1.728^***^ | 2.846^***^ | 97.405^**^ |
|  | (0.260) | (0.093) | (39.609) |
| N | 3715 | 2791 | 2685 |
| R-sq | 0.1 | 0.057 | 0.022 |

Note. ^*^, ^**^, ^***^ corresponding to p values ≤ 0.10, ≤ 0.05 and ≤ 0.01, respectively . 95% confidence interval reported in brackets.
