# Supplementary material for: How did the urban and rural resident basic medical insurance integration affect medical costs?—Evidence from China
Source: PLoS One. 2025 Jul 18;20(7):e0325614. doi: 10.1371/journal.pone.0325614 (PMC12274002; doi:10.1371/journal.pone.0325614)
Supplement: S15 Table — (DOCX) [file pone.0325614.s015.docx]

**S15 Table.** Impact of URRBMI integration on health awareness and preventive behaviors

|  | Outpatient type | Inpatient type | Distance to medical institutions |
| --- | --- | --- | --- |
| DID | 0.193^***^ | -0.357^***^ | 0.060^***^ |
|  | (0.019) | (0.012) | (0.014) |
| Age | -0.004^***^ | -0.001^*^ | 0.009^***^ |
|  | (0.001) | 0.000 | (0.001) |
| Sex | -0.011 | 0.232^***^ | -0.026^**^ |
|  | (0.008) | (0.006) | (0.010) |
| Marriage | 0.007 | -0.032^***^ | 0.016 |
|  | (0.013) | (0.006) | (0.016) |
| Regular medical checkups | 0.031^***^ | -0.015^***^ |  |
|  | (0.009) | (0.006) |  |
| Health Status | 0.016^***^ | 0.000 | -0.010^***^ |
|  | (0.003) | (0.002) | (0.003) |
| Disability | 0.003 | -0.010^*^ | 0.044^***^ |
|  | (0.011) | (0.006) | (0.009) |
| Drinking | 0.041^***^ |  | -0.004 |
|  | (0.008) |  | (0.009) |
| Smoking | -0.080^***^ |  | -0.033^***^ |
|  | (0.018) |  | (0.013) |
| Income | 0.001 | 0.005^*^ | 0 |
|  | (0.003) | (0.002) | (0.004) |
| Time effect | YES | YES | YES |
| Region effect | YES | YES | YES |
| cons | 0.926^***^ | 0.129^**^ | -0.092 |
|  | (0.088) | (0.056) | (0.078) |
| N | 17583 | 21057 | 21047 |
| R-sq | 0.07 | 0.322 | 0.077 |

Note. ^*^, ^**^, ^***^ corresponding to p values ≤ 0.10, ≤ 0.05 and ≤ 0.01, respectively . 95% confidence interval reported in brackets.
