# Supplementary material for: How did the urban and rural resident basic medical insurance integration affect medical costs?—Evidence from China
Source: PLoS One. 2025 Jul 18;20(7):e0325614. doi: 10.1371/journal.pone.0325614 (PMC12274002; doi:10.1371/journal.pone.0325614)
Supplement: S16 Table — (DOCX) [file pone.0325614.s016.docx]

**S16 Table.** Joint significance test for leads and lags

|  | leads | lags |
| --- | --- | --- |
| F-stat | 1.534 | 85.978 |
| P-value | 0.207 | 0.000 |
| Degrees of freedom | (6,26) | (3,26) |

*Note.* The above equilibrium trend test was performed using event study methodology
