# Supplementary material for: How did the urban and rural resident basic medical insurance integration affect medical costs?—Evidence from China
Source: PLoS One. 2025 Jul 18;20(7):e0325614. doi: 10.1371/journal.pone.0325614 (PMC12274002; doi:10.1371/journal.pone.0325614)
Supplement: S17 Table — (DOCX) [file pone.0325614.s017.docx]

**S17 Table.** Impact of URRBMI integration on healthcare resource utilization and medical costs for residents over 65 years of age

|  | Outpatient  visits | Inpatient  visits | Outpatient OOP  costs | Inpatient OOP costs | Medical expenditure |
| --- | --- | --- | --- | --- | --- |
| DID | 0.001 | -0.02 | 0.113 | 0.342^*^ | 0.352^***^ |
|  | (0.003) | (0.028) | (0.117) | (0.179) | (0.100) |
| Age | 0.000 | 0.006^***^ | -0.003 | -0.012 | 0.006 |
|  | (0.000) | (0.001) | (0.010) | (0.010) | (0.008) |
| Sex | 0.001 | 0.022^**^ | 0.090 | -0.14 | -0.036 |
|  | (0.002) | (0.011) | (0.127) | (0.128) | (0.075) |
| Marriage | -0.002 | -0.019 | -0.009 | 0.048 | 0.473^***^ |
|  | (0.002) | (0.017) | (0.122) | (0.144) | (0.094) |
| Regular medical checkups | 0.005^***^ | 0.034^**^ | -0.106 | -0.088 | 0.118 |
|  | (0.002) | (0.014) | (0.104) | (0.104) | (0.094) |
| Health Status | 0 | -0.080^***^ | -0.132 | -0.258^***^ | 0.154 |
|  | (0.001) | (0.005) | (0.080) | (0.039) | (0.104) |
| Disability | 0.051^***^ | 0.081^***^ | 0.254 | -0.029 | 0.249^**^ |
|  | (0.009) | (0.020) | (0.204) | (0.146) | (0.096) |
| Drinking | -0.001 | -0.057^***^ | -0.186 | -0.407^**^ | -0.244^**^ |
|  | (0.002) | (0.010) | (0.141) | (0.176) | (0.097) |
| Smoking | -0.002 | -0.080^***^ | -0.152 | -0.223 | -0.115 |
|  | (0.003) | (0.026) | (0.221) | (0.223) | (0.117) |
| Income | -0.001 | 0.009^*^ | 0.04 | 0.155^***^ | 0.002 |
|  | (0.001) | (0.006) | (0.038) | (0.045) | (0.020) |
| Time effect | YES | YES | YES | YES | YES |
| Region effect | YES | YES | YES | YES | YES |
| _cons | -0.015 | -0.031 | 6.414^***^ | 10.059^***^ | 8.441^***^ |
|  | (0.014) | (0.117) | (0.955) | (0.887) | (0.583) |
| N | 7970 | 7963 | 680 | 737 | 2206 |
| R-sq | 0.044 | 0.098 | 0.251 | 0.306 | 0.096 |

Note. ^*^, ^**^, ^***^ corresponding to p values ≤ 0.10, ≤ 0.05 and ≤ 0.01, respectively . 95% confidence interval reported in brackets.
