# Supplementary material for: How did the urban and rural resident basic medical insurance integration affect medical costs?—Evidence from China
Source: PLoS One. 2025 Jul 18;20(7):e0325614. doi: 10.1371/journal.pone.0325614 (PMC12274002; doi:10.1371/journal.pone.0325614)
Supplement: S18 Table — (DOCX) [file pone.0325614.s018.docx]

**S18 Table.** Impact of URRBMI integration on healthcare resource utilization and medical costs for residents under 65 years of age

|  | Outpatient visits | Inpatient visits | Outpatient OOP costs | Inpatient OOP costs | Medical expenditure |
| --- | --- | --- | --- | --- | --- |
| DID | -0.002 | 0.038^***^ | 0.187^*^ | 0.432^**^ | 0.382^***^ |
|  | (0.003) | (0.012) | (0.112) | (0.191) | (0.101) |
| Age | 0.000 | 0.004^***^ | -0.008 | -0.015 | 0.006 |
|  | (0.000) | (0.001) | (0.010) | (0.010) | (0.008) |
| Sex | 0.000 | 0.030^***^ | 0.149 | -0.088 | -0.036 |
|  | (0.002) | (0.007) | (0.118) | (0.126) | (0.076) |
| Marriage | -0.002 | -0.012 | -0.05^*^ | 0.06 | 0.469^***^ |
|  | (0.003) | (0.009) | (0.119) | (0.137) | (0.097) |
| Regular medical checkups | 0.005^***^ | 0.052^***^ | -0.085 | -0.138 | 0.128 |
|  | (0.002) | (0.008) | (0.103) | (0.104) | (0.093) |
| Health Status | 0.000 | -0.079^***^ | -0.158^*^ | 0.171 | -0.254^***^ |
|  | (0.001) | (0.004) | (0.080) | (0.104) | (0.040) |
| Disability | 0.053^***^ | 0.091^***^ | 0.342^*^ | -0.014 | 0.278^***^ |
|  | (0.009) | (0.017) | (0.199) | (0.142) | (0.101) |
| Drinking | -0.001 | -0.048^***^ | -0.136 | -0.396^**^ | -0.250^**^ |
|  | (0.002) | (0.016) | (0.143) | (0.175) | (0.098) |
| Smoking | -0.003 | -0.086 | -0.086 | -0.178 | -0.126 |
|  | (0.002) | (0.207) | (0.207) | (0.212) | (0.113) |
| Income | -0.001 | -0.002 | 0.019 | 0.117^***^ | -0.001 |
|  | (0.001) | (0.003) | (0.037) | (0.042) | (0.020) |
| Time effect | YES | YES | YES | YES | YES |
| Region effect | YES | YES | YES | YES | YES |
| _cons | -0.018 | 0.104 | 6.846^***^ | 10.308^***^ | 8.465^***^ |
|  | (0.015) | (0.091) | (0.970) | (0.897) | (0.598) |
| N | 7724 | 9929 | 628 | 641 | 2200 |
| R-sq | 0.046 | 0.076 | 0.196 | 0.165 | 0.097 |

Note. ^*^, ^**^, ^***^ corresponding to p values ≤ 0.10, ≤ 0.05 and ≤ 0.01, respectively . 95% confidence interval reported in brackets.
