# Supplementary material for: How did the urban and rural resident basic medical insurance integration affect medical costs?—Evidence from China
Source: PLoS One. 2025 Jul 18;20(7):e0325614. doi: 10.1371/journal.pone.0325614 (PMC12274002; doi:10.1371/journal.pone.0325614)
Supplement: S19 Table — (DOCX) [file pone.0325614.s019.docx]

**S19 Table.** Impact of URRBMI integration on type of medical institutions and distance to medical institutions for residents over 65 years of age

|  | Type of outpatient | Type of inpatient | Distance to medical institutions |
| --- | --- | --- | --- |
| DID | 0.129^***^ | 0.070^*^ | 35.937^***^ |
|  | (0.047) | (0.037) | (13.693) |
| Age | -0.002 | 0.001 | -1.154^**^ |
|  | (0.003) | (0.003) | (0.529) |
| Sex | 0.076^*^ | 0.007 | -9.508 |
|  | (0.039) | (0.024) | (12.228) |
| Marriage | 0.069 | -0.021 | 16.682^*^ |
|  | (0.048) | (0.029) | (8.967) |
| Regular medical checkups | 0.063 | -0.034 | -9.231 |
|  | (0.039) | (0.027) | (11.854) |
| Health Status | -0.026 | -0.021 | -4.576 |
|  | (0.021) | (0.014) | (3.516) |
| Disability | 0.170^***^ | 0.038 | 17.303 |
|  | (0.041) | (0.048) | (19.266) |
| Drinking | -0.165^***^ | -0.095^***^ | 0.899 |
|  | (0.044) | (0.033) | (6.147) |
| Smoking | -0.06 | 21.091 | 0.004 |
|  | (0.062) | (14.478) | (0.190) |
| Income | -0.009 | 0.016 | -2.356 |
|  | (0.014) | (0.012) | (1.714) |
| Time effect | YES | YES | YES |
| Region effect | YES | YES | YES |
| _cons | 1.832^***^ | 2.930^***^ | 103.438^***^ |
|  | (0.292) | (0.190) | (38.839) |
| N | 2005 | 1442 | 1373 |
| R-sq | 0.091 | 0.088 | 0.023 |

Note. ^*^, ^**^, ^***^ corresponding to p values ≤ 0.10, ≤ 0.05 and ≤ 0.01, respectively . 95% confidence interval reported in brackets.
