# Supplementary material for: How did the urban and rural resident basic medical insurance integration affect medical costs?—Evidence from China
Source: PLoS One. 2025 Jul 18;20(7):e0325614. doi: 10.1371/journal.pone.0325614 (PMC12274002; doi:10.1371/journal.pone.0325614)
Supplement: S20 Table — (DOCX) [file pone.0325614.s020.docx]

**S20 Table.** Impact of URRBMI integration on type of medical institutions and distance to medical institutions for residents under 65 years of age

|  | Type of outpatient | Type of inpatient | Distance to medical institutions |
| --- | --- | --- | --- |
| DID | 0.274^***^ | 0.049 | 25.329^**^ |
|  | (0.057) | (0.042) | (10.676) |
| Age | -0.002 | 0.002 | -0.892 |
|  | (0.003) | (0.002) | (0.937) |
| Sex | 0.05 | 0.018 | 7.635 |
|  | (0.046) | (0.027) | (8.729) |
| Marriage | 0.024 | 0.069^*^ | -2.773 |
|  | (0.065) | (0.041) | (26.517) |
| Regular medical checkups | 0.226^***^ | -0.01 | 15.871 |
|  | (0.039) | (0.028) | (11.840) |
| Health Status | 0.01 | 0.001 | -2.767 |
|  | (0.019) | (0.012) | (5.266) |
| Disability | 0.229^***^ | -0.036 | -1.099 |
|  | (0.033) | (0.048) | (22.469) |
| Drinking | -0.087^*^ | -0.006 | -1.101 |
|  | (0.045) | (0.035) | (11.689) |
| Smoking | -0.129^**^ | -0.063 | 10.208 |
|  | (0.060) | (0.051) | (12.483) |
| Income | 0.023 | 0.022^**^ | 0.491 |
|  | (0.015) | (0.010) | (3.668) |
| Time effect | YES | YES | YES |
| Region effect | YES | YES | YES |
| _cons | 1.878^***^ | 2.692^***^ | 91.256 |
|  | (0.243) | (0.146) | (82.058) |
| N | 2293 | 1349 | 1312 |
| R-sq | 0.126 | 0.027 | 0.035 |

Note. ^*^, ^**^, ^***^ corresponding to p values ≤ 0.10, ≤ 0.05 and ≤ 0.01, respectively . 95% confidence interval reported in brackets.
