# Supplementary material for: How did the urban and rural resident basic medical insurance integration affect medical costs?—Evidence from China
Source: PLoS One. 2025 Jul 18;20(7):e0325614. doi: 10.1371/journal.pone.0325614 (PMC12274002; doi:10.1371/journal.pone.0325614)
Supplement: S21 Table — (DOCX) [file pone.0325614.s021.docx]

**S21 Table.** Impact of URRBMI integration on health awareness and preventive behaviors for residents over 65 years of age

|  | Physical exercise | Smoking | Regular medical checkups |
| --- | --- | --- | --- |
| DID | 0.253^***^ | -0.363^***^ | 0.110^***^ |
|  | (0.033) | (0.016) | (0.028) |
| Age | -0.007^***^ | -0.002^**^ | 0.003^***^ |
|  | (0.001) | (0.001) | (0.001) |
| Sex | -0.012 | 0.179^***^ | -0.024 |
|  | (0.010) | (0.008) | (0.015) |
| Marriage | -0.01 | -0.033^***^ | 0.023 |
|  | (0.016) | (0.008) | (0.020) |
| Regular medical checkups | 0.045^***^ | -0.017^**^ |  |
|  | (0.013) | (0.008) |  |
| Health Status | 0.024^***^ | 0.005 | -0.009 |
|  | (0.006) | (0.003) | (0.006) |
| Disability | -0.022 | 0.009 | 0.031^*^ |
|  | (0.018) | (0.011) | (0.018) |
| Drinking | 0.063^***^ |  | 0.001 |
|  | (0.014) |  | (0.009) |
| Smoking | -0.035 |  | -0.032^**^ |
|  | (0.031) |  | (0.012) |
| Income | 0.012^**^ | 0.004 | 0.005 |
|  | (0.005) | (0.003) | (0.006) |
| Region effect | YES | YES | YES |
| Time effect | YES | YES | YES |
| _cons | 1.402^***^ | 0.100^*^ | 0.237 |
|  | (0.100) | (0.059) | (0.198) |
| N | 6860 | 7973 | 7973 |
| R-sq | 0.093 | 0.3 | 0.057 |

Note. ^*^, ^**^, ^***^ corresponding to p values ≤ 0.10, ≤ 0.05 and ≤ 0.01, respectively . 95% confidence interval reported in brackets.
