# Supplementary material for: How did the urban and rural resident basic medical insurance integration affect medical costs?—Evidence from China
Source: PLoS One. 2025 Jul 18;20(7):e0325614. doi: 10.1371/journal.pone.0325614 (PMC12274002; doi:10.1371/journal.pone.0325614)
Supplement: S22 Table — (DOCX) [file pone.0325614.s022.docx]

**S22 Table.** Impact of URRBMI integration on health awareness and preventive behaviors for residents under 65 years of age

|  | Physical exercise | Smoking | Regular medical checkups |
| --- | --- | --- | --- |
| DID | 0.159^***^ | -0.355^***^ | 0.031^***^ |
|  | (0.017) | (0.012) | (0.011) |
| Age | -0.003^***^ | -0.001 | 0.003^***^ |
|  | (0.001) | 0.000 | (0.001) |
| Sex | -0.008 | 0.265^***^ | -0.025^***^ |
|  | (0.009) | (0.007) | (0.009) |
| Marriage | 0.018 | -0.014 | 0.006 |
|  | (0.017) | (0.009) | (0.015) |
| Regular medical checkups | 0.01 | -0.015^**^ |  |
|  | (0.010) | (0.007) |  |
| Health Status | 0.011^***^ | -0.003 | -0.010^***^ |
|  | (0.004) | (0.003) | (0.003) |
| Disability | 0.02 | -0.018^**^ | 0.051^***^ |
|  | (0.013) | (0.008) | (0.011) |
| Drinking | 0.025^**^ |  | 0.050^*^ |
|  | (0.010) |  | (0.015) |
| Smoking | -0.100^***^ |  | -0.089^***^ |
|  | (0.019) |  | (0.025) |
| Income | -0.005^*^ | 0.006^**^ | 0.001 |
|  | (0.003) | (0.003) | (0.003) |
| Region effect | YES | YES | YES |
| Time effect | YES | YES | YES |
| _cons | 0.802^***^ | 0.126^*^ | 0.227^***^ |
|  | (0.124) | (0.068) | (0.071) |
| N | 10723 | 13084 | 13077 |
| R-sq | 0.062 | 0.339 | 0.049 |

Note. ^*^, ^**^, ^***^ corresponding to p values ≤ 0.10, ≤ 0.05 and ≤ 0.01, respectively . 95% confidence interval reported in brackets.
