# Supplementary material for: How did the urban and rural resident basic medical insurance integration affect medical costs?—Evidence from China
Source: PLoS One. 2025 Jul 18;20(7):e0325614. doi: 10.1371/journal.pone.0325614 (PMC12274002; doi:10.1371/journal.pone.0325614)
Supplement: S23 Table — (DOCX) [file pone.0325614.s023.docx]

**S23 Table.** Impact of URRBMI integration on induced demand- K-nearest neighbor matching (1:2 ratio)

|  | Outpatient OOP Costs | Inpatient OOP Costs | Medical expenditure |
| --- | --- | --- | --- |
| DID | 0.270^***^ | 0.282^**^ | 0.384^***^ |
|  | (0.065) | (0.133) | (0.060) |
| Age | 0.002 | -0.005 | 0.004 |
|  | (0.003) | (0.005) | (0.003) |
| Sex | 0.061 | 0.043 | 0.027 |
|  | (0.067) | (0.104) | (0.053) |
| Marriage | 0.115 | 0.138 | 0.353^***^ |
|  | (0.086) | (0.130) | (0.074) |
| Regular medical checkups | 0.204^***^ | -0.11 | 0.194^***^ |
|  | (0.066) | (0.094) | (0.050) |
| Health Status | 0.342^***^ | -0.205^***^ | -0.263^***^ |
|  | (0.049) | (0.053) | (0.024) |
| Disability | 0.478^***^ | -0.184 | 0.134^**^ |
|  | (0.092) | (0.118) | (0.067) |
| Drinking | -0.259^***^ | -0.171 | -0.197^***^ |
|  | (0.081) | (0.137) | (0.057) |
| Smoking | 0.000 | -0.136 | -0.09 |
|  | (0.000) | (0.203) | (0.089) |
| Income | 0.047^**^ | 0.093^***^ | 0.030^*^ |
|  | (0.022) | (0.035) | (0.017) |
| Satisfaction with medical services | -0.078^***^ | -0.152^***^ | -0.051^**^ |
|  | (0.028) | (0.042) | (0.021) |
| Region effect | YES | YES | YES |
| Time effect | YES | YES | YES |
| _cons | 4.456^***^ | 10.233^***^ | 7.664^***^ |
|  | (0.501) | (0.769) | (0.314) |
| N | 2307 | 927 | 4593 |
| R-sq | 0.132 | 0.303 | 0.069 |

Note. ^*^, ^**^, ^***^ corresponding to p values ≤ 0.10, ≤ 0.05 and ≤ 0.01, respectively . 95% confidence interval reported in brackets.
