# Supplementary material for: How did the urban and rural resident basic medical insurance integration affect medical costs?—Evidence from China
Source: PLoS One. 2025 Jul 18;20(7):e0325614. doi: 10.1371/journal.pone.0325614 (PMC12274002; doi:10.1371/journal.pone.0325614)
Supplement: S24 Table — (DOCX) [file pone.0325614.s024.docx]

**S24 Table.** Policy effects of URRBMI integration after removing the impact of the COVID-19

|  | Outpatient visits | Inpatient visits | Outpatient OOP costs | Inpatient OOP costs | Medical expenditure |
| --- | --- | --- | --- | --- | --- |
| DID | 0.000 | 0.019^*^ | 0.216^**^ | 0.238^*^ | 1.664^***^ |
|  | (0.002) | (0.011) | (0.084) | (0.121) | (0.626) |
| Age | 0.000 | 0.004^***^ | -0.005 | -0.002 | -0.024 |
|  | (0.000) | (0.001) | (0.004) | (0.005) | (0.034) |
| Sex | 0.001 | 0.022^**^ | 0.031 | 0.033 | 0.093 |
|  | (0.001) | (0.009) | (0.072) | (0.097) | (0.379) |
| Marriage | 0.000 | -0.021 | 0.105 | 0.122 | 2.006^**^ |
|  | (0.002) | (0.016) | (0.115) | (0.117) | (0.867) |
| Regular medical checkups | 0.008^***^ | 0.049^***^ | 0.026 | 0.007 | 0.247 |
|  | (0.001) | (0.012) | (0.072) | (0.083) | (0.519) |
| Health status | 0.000 | -0.072^***^ | -0.135^***^ | -0.876^**^ | 0.244^***^ |
|  | (0.001) | (0.003) | (0.050) | (0.425) | (0.073) |
| Disability | 0.052^***^ | 0.069^***^ | 0.256^**^ | -0.161^*^ | 0.543 |
|  | (0.005) | (0.010) | (0.119) | (0.094) | (0.900) |
| Drinking | 0.000 | -0.028^***^ | -0.257^**^ | -0.301^***^ | -0.126 |
|  | (0.001) | (0.009) | (0.109) | (0.106) | (0.542) |
| Smoking | -0.001 | -0.045^***^ | -0.088 | -0.362^**^ | -1.174 |
|  | (0.002) | (0.011) | (0.126) | (0.158) | (0.740) |
| Income | 0.000 | 0.008 | 0.045^*^ | 0.117^***^ | -2.448^***^ |
|  | (0.000) | (0.005) | (0.025) | (0.033) | (0.284) |
| Time effect | YES | YES | YES | YES | YES |
| Region effect | YES | YES | YES | YES | YES |
| _cons | -0.004 | 0.09 | 5.822^***^ | 8.429^***^ | 15.932^***^ |
|  | (0.006) | (0.069) | (0.410) | (0.501) | (2.846) |
| N | 21047 | 21033 | 1710 | 1266 | 4676 |
| R-sq | 0.038 | 0.077 | 0.14 | 0.229 | 0.044 |

Note. ^*^, ^**^, ^***^ corresponding to p values ≤ 0.10, ≤ 0.05 and ≤ 0.01, respectively . 95% confidence interval reported in brackets.
