# Supplementary figures and images for: How did the urban and rural resident basic medical insurance integration affect medical costs?—Evidence from China
Source: PLoS One. 2025 Jul 18;20(7):e0325614. doi: 10.1371/journal.pone.0325614 (PMC12274002; doi:10.1371/journal.pone.0325614)

**S1 Fig.** Sample selection and inclusion/exclusion criteria


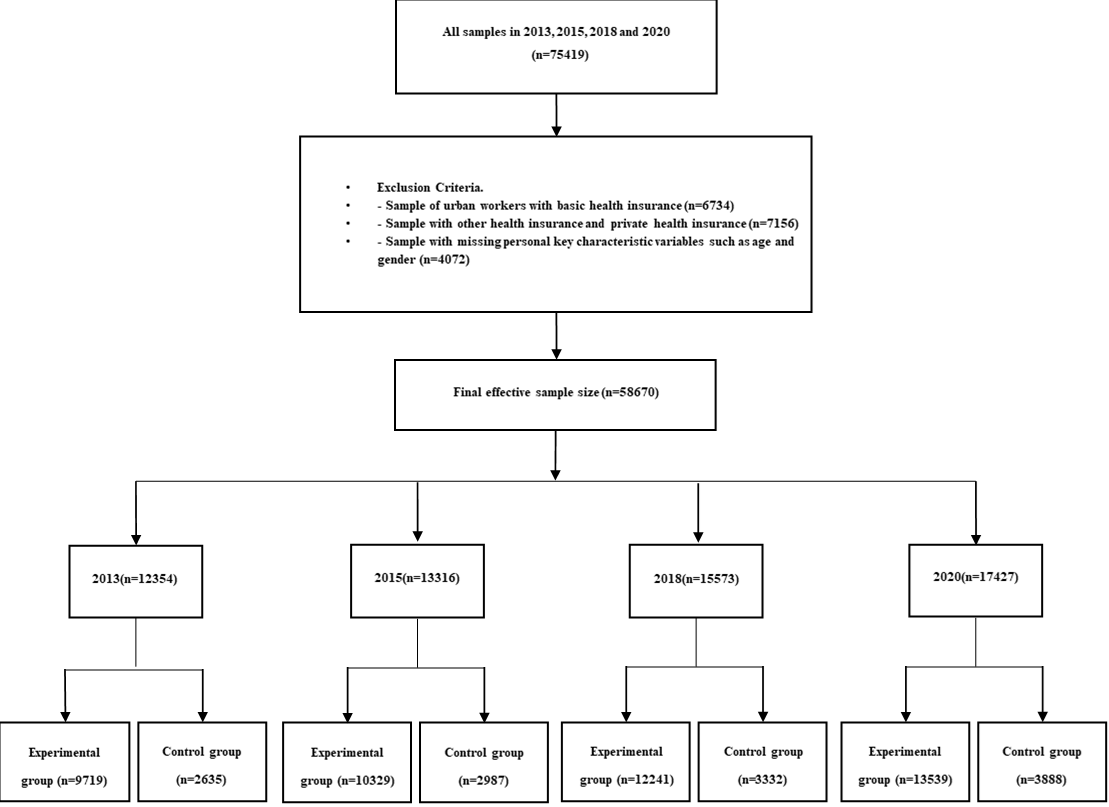

Supplement: S1 Fig — (DOCX) [file pone.0325614.s025.docx]

**S2 Fig. Degree of control variable dispersion before and after PSM matching**

**
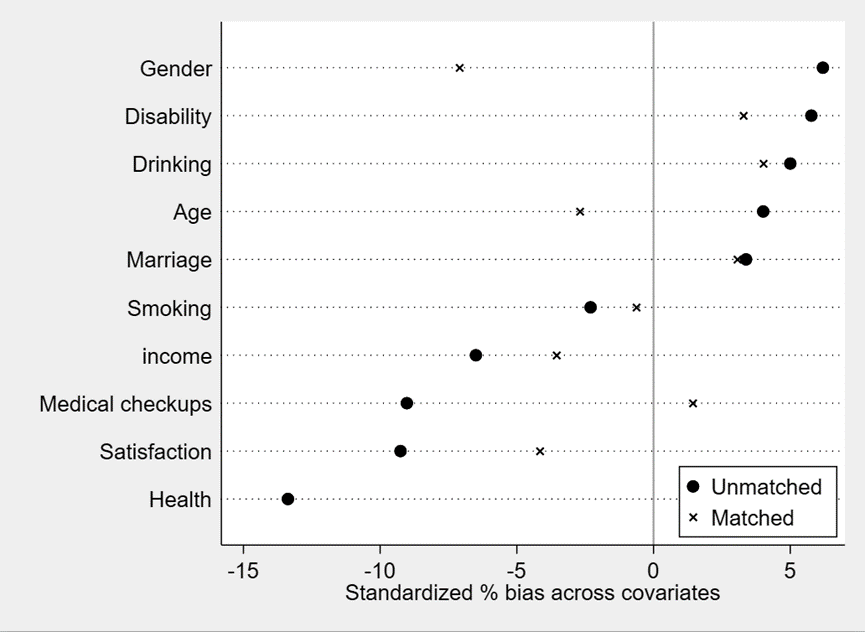
**

Supplement: S2 Fig — (DOCX) [file pone.0325614.s026.docx]

**S3 Fig.** **Degree of control variable dispersion before and after PSM matching**


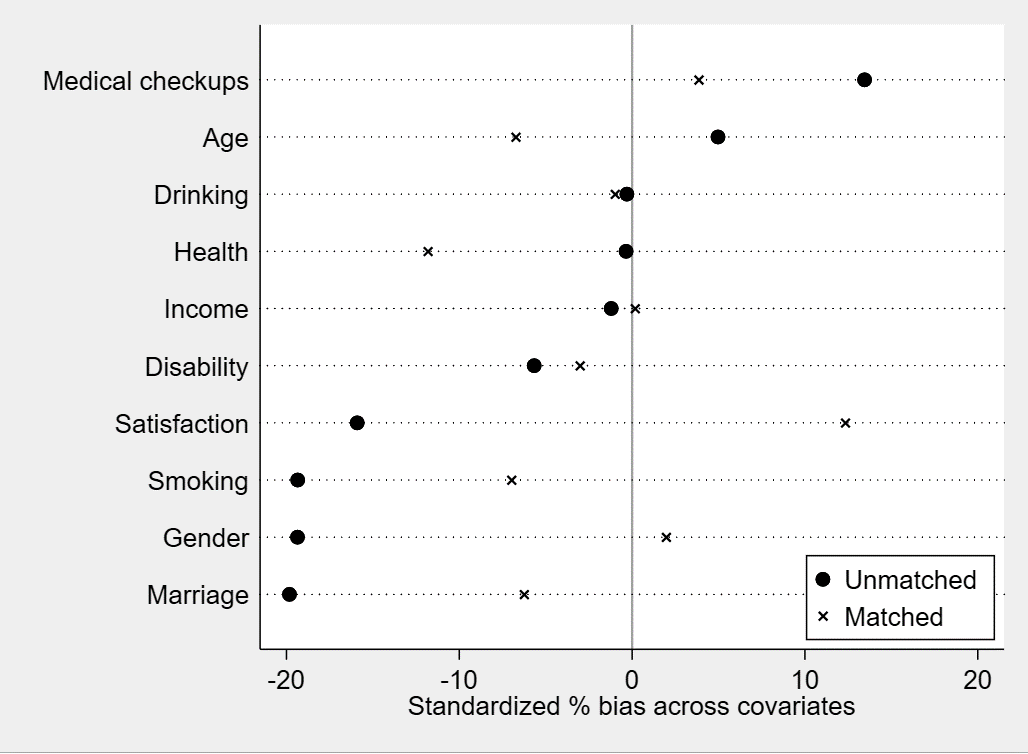

Supplement: S3 Fig — (DOCX) [file pone.0325614.s027.docx]

**S4 Figure. Degree of control variable dispersion before and after PSM matching**


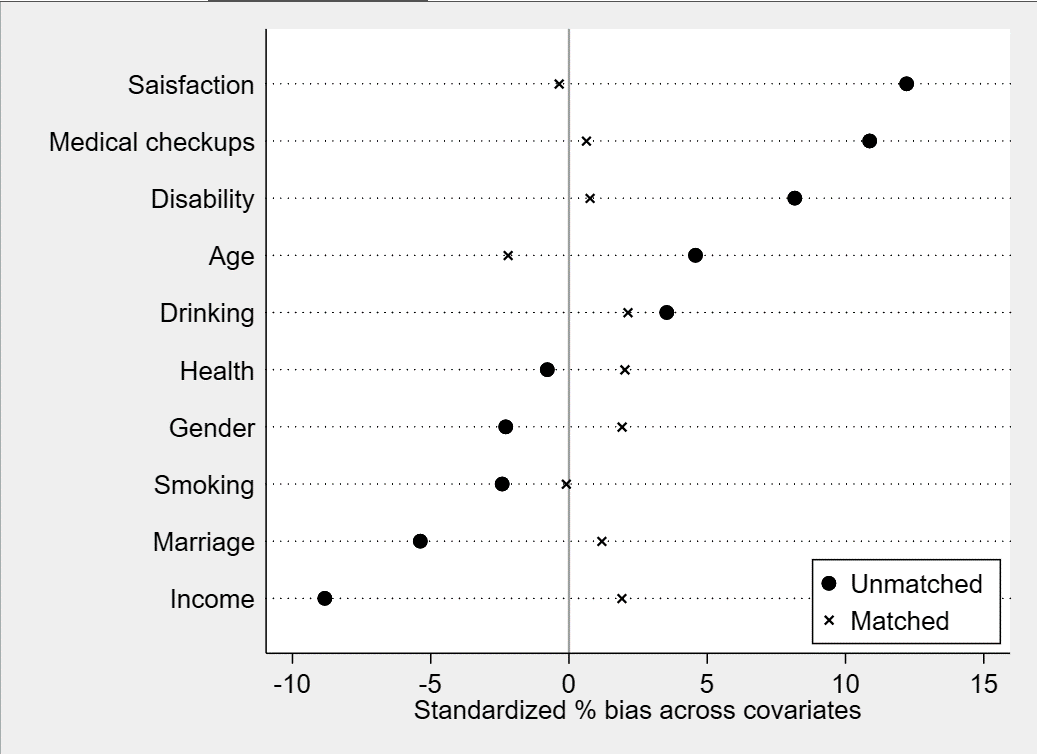

Supplement: S4 Fig — (DOCX) [file pone.0325614.s028.docx]

**S5 Fig.** Equilibrium trend test of medical costs

**
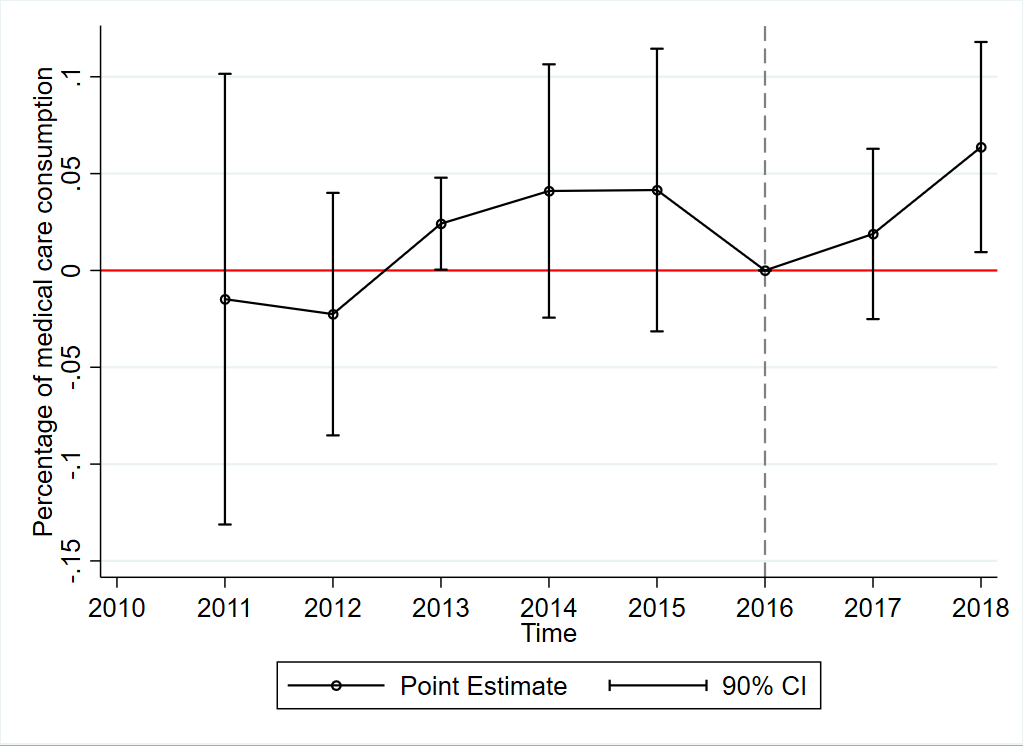
**

Supplement: S5 Fig — (DOCX) [file pone.0325614.s029.docx]
